# Wounds of the Peritoneum

**Published:** 1898-04-16

**Authors:** 


### Editor's Letter-Box.

[Our Correspondents are reminded that prolixity is a great bar to publication, and that brevity of style and conciseness of statement greatly facilitate early insertion.]

### WOUNDS OF THE PERITONEUM.

Mr. John Ewens, Consulting Surgeon to the Bristol Royal Hospital for Sick Children and Women, writes: I think the following case may be interesting as bearing upon those reported in your journal of to-day in reference to penetrating wounds of the abdomen. About thirty-five years ago I was hurriedly summoned to a man who, whilst grinding his scythe, allowed it to slip, the point penetrating the epigastrium, and inflicting a wound about one and a half inches in length. He was much collapsed and frightened, but in no pain. The cleansed finger (this was before antiseptic surgery was known) introduced into the wound easily detected the full stomach (about one hour after a hearty breakfast), the liver, colon, &c., all intact. By means of two or three sutures through the abdominal walls (peritoneum included) perfect apposition was effected; a few doses of opium were given; primary union occurred without pain or discomfort. At the end of the week I found the man actively engaged in controlling his brother struggling in a severe epileptic fit. Some years after, curiously enough, the same man or his brother—at this distance of time I forget which—fell on his scythe when returning from mowing, and cut completely through the lig. patellæ, exposing the knee-joint fully. My friend Dr. Fielding, of Milton Abbas, Dorset, saw the patient at once, and having thoroughly cleansed the parts with carbolic acid lotion, brought the edges of the wound and ligament together and put on a long back-splint with antiseptic dressings. Primary union resulted, and on the eighteenth day the man—of course, contrary to orders—walked about for three days, at the end of which, when intoxicated, he fell down, tore open the recently-united parts, and, I need scarcely add, severe inflammation with suppuration intervened, and the man barely escaped with his life. After protracted suffering and suppuration amongst the thigh muscles, he at last recovered with a stiff knee, and resumed work in due course. I saw him very soon after the first injury to the knee, and was astonished at the result. I also saw him frequently during the later period, and was equally astonished at his recovery.

